# PReS-FINAL-2189: Tocillizumab for patients with takayasu arteritis in childhood refractory to conventional therapy

**DOI:** 10.1186/1546-0096-11-S2-O24

**Published:** 2013-12-05

**Authors:** K Yamazaki, M Kikuchi, T Nozawa, T Kanetaka, R Hara, T Imagawa, K Nishimura, N Sakurai, T Sato, S Yokota

**Affiliations:** 1 Pediatrics, Yokohama City University School of Medicine, yokohama, Japan

## Introduction

Takayasu arteritis (TA) is a chronic systemic granulomatous vasculitis of large vessels. Corticosteroids may induce remission. However, over half of patients flare with tapering of corticosteroids. There are anecdotal reports of treatment with methotrexate, azathioprine, and biologics such as infliximab. Cyclophosphamide is recommended for life-threatening or organ-threatening patients. However, patients, especially with HLA B52-positive, are frequently refractory to these treatments. Since serum levels of interleukin (IL)-6 are correlated with disease activity, an open-label trial of tocilizumab (TCZ), an anti-IL-6 receptor monoclonal antibody, was conducted for 6 pediatric patients with TA.

## Objectives

To assess the efficacy and safety of TCZ for patients with TA refractory to conventional treatments.

## Methods

Six patients with TA were eligible in this study. Diagnosis of TA was made by clinical manifestations, blood examinations and imaging assessments. TCZ (8-10 mg/kg) was intravenously administered in 6 patients with TA every 2 weeks. The efficacy and tolerability of TCZ were evaluated by TA disease activity, laboratory findings, and corticosteroid reductions at 6, 12 and 24 months after TCZ initiation. Ultrasound, [^18^F]-FDG-PET, PET/CT, and MRI angiogram were performed before and after TCZ treatment.

## Results

The median onset age was 12.5 years (range; 7-17 years) and the median disease duration was 94 months (range; 18-192 months). Among 6 patients with TA, there was 1 patient complicated with ulcerative colitis, 1 patient with heart failure, and 1 patient with brain ischemia. HLA-B52 was positive in 4 out of 6 patients. The previous treatments, which were judged as ineffective or had side effects, were corticosteroids (6/6), intravenous cyclophosphamide (5/6), azathioprine (3/6), mycophenolate mofetil (2/6), methotrexate (2/6) and infliximab (2/6). The mean duration of TCZ treatment was 24 months (range to 6-58 months). Before starting TCZ, patients had clinical symptoms of fever (2/6), myalgia (4/6), headache (4/6), hypertension (2/6) and abdominal pain (1/6). All of these symptoms were improved during TCZ therapy. The mean erythrocyte sedimentation rate was 36.1 mm/hour (range 11-58 mm/hour) before TCZ, and it decreased to 3.2 mm/hour (range 1-6 mm/hour) 6 months after TCZ. The mean doses of prednisolone were 24.3 mg/day (range 11-45 mg/day) before TCZ, and they decreased to 18.0 mg/day (range 11-40 mg/day), 14.1 mg/day (range 10-22.5 mg/day), and 5.7 mg/day (range 5-6.5 mg/day) in 6, 12 and 24 months, respectively, after starting TCZ. Although serum IL-6 levels increased in 2-3 months after the initial TCZ administration with the mean level of 198.8 pg/ml (range 20.7-548.1 pg/ml), they gradually decreased to 33.3 pg/ml (range 7.1-54.8 pg/ml) within 6 months. MRI angiogram findings were improved in 3/6 cases 6 months to 2 years after TCZ therapy. No serious adverse events were observed.

## Conclusion

TCZ was effective and well tolerated for patients with TA refractory to corticosteroids and various immunosupressants.

## Disclosure of interest

None declared.

